# Characteristics Analysis and Identification of Key Sectors of Air Pollutant Emissions in China from the Perspective of Complex Metabolic Network

**DOI:** 10.3390/ijerph19159396

**Published:** 2022-07-31

**Authors:** Jiekun Song, Lina Jiang, Zeguo He, Zhicheng Liu, Xueli Leng

**Affiliations:** School of Economics and Management, China University of Petroleum, Qingdao 266580, China; z21080318@s.upc.edu.cn (Z.H.); z22080013@s.upc.edu.cn (Z.L.); 201801080108@sdust.edu.cn (X.L.)

**Keywords:** air pollutants, environment input–output model, complex network, metabolic network, key sectors

## Abstract

Presently, China is in a critical period of economic transformation and upgrading. At the same time, it is also facing the pressure of serious atmospheric environmental pollution, which seriously threatens human health and hinders the sustainable economic development. Air pollutants are closely related to economic sectors, which together constitute a complex network. Air pollutants form an input–output ecological metabolic relationship among different sectors. Therefore, from the perspective of complex metabolic network, this study first constructs an environmental input–output model and then comprehensively uses the relevant methods of ecological network analysis and complex network analysis to analyze the characteristics of China’s air pollutant emission system. Secondly, the key joint sectors of NO_x_ and PM emissions are determined from the supply side and the demand side, respectively. Finally, the corresponding emission reduction measures are proposed for the identified key sectors.

## 1. Introduction

Due to the rapid development of industry, the continuous expansion of urbanization and the huge consumption of fossil energy, China has been suffering from serious air pollution in the past few years, which has widely affected the environment and human health [1,2]. In order to improve air quality and promote the prevention and control of air pollution, the Chinese government has made great efforts, such as implementing the “Air Pollution Prevention and Control Action Plan” (2013–2017) and the “Blue Sky Protection Campaign” (2018–2020), and achieved remarkable results. Total emissions of sulfur dioxide (SO_2_), nitrogen oxide (NO_x_), particulate matter (PM) and other major pollutants continued to decline [3,4,5]. However, according to the statistics of the Ministry of Ecology and Environment of the Peoples Republic of China [6], the air quality in 121 cities still failed to meet the ambient air quality standards in 2021, accounting for 35.7% of all the monitored cities. The main reason for the failure was that the annual average concentration of particulate matter in these cities exceeded the standards specified in the WHO global air quality guidelines [7].

As the by-products of economic production, the emissions of air pollutants are closely related to the production activities of various economic sectors [8,9]. On the one hand, agriculture, industry and other economic sectors directly discharge air pollutants in the production process [10,11]. On the other hand, indirect emissions are embodied in the circulation of intermediate products in the industrial chain [12,13], that is, as products are consumed by other sectors, the pollutants produced in the production process are also implicitly discharged among other sectors. At the same time, the products of other sectors are consumed by all sectors, including the production sector, thus forming a complex metabolic network of pollutant emissions. From the perspective of complex network and metabolic network, analyzing the embodied air pollutant emissions of each sector and the correlation between sectors will help us to grasp the characteristics of the whole air pollutant emission system, analyze the key source sectors and provide a decision-making basis for air pollutant emission reduction.

The input–output analysis (IOA) model, based on a theory of linear algebra, can adequately depict the linkage between intersectoral production technologies and final demand patterns [14]. Considering different ecological elements, scholars extended the basic IOA model and proposed energy IOA model [15,16], water IOA model [17,18], environmental IOA (EIOA) model [19,20], etc. The EIOA model covers all elements related to the environment, and it can be used to disaggregate direct and indirect pollutant emissions and calculate the embodied pollutant emissions in economic sectors. Huo et al. [21] applied the EIOA model to calculate the embodied pollutant emissions and found that the equipment, machinery and devices manufacturing and construction sectors contributed to more than 50% of SO_2_, NO_x_ and PM_2.5_ emissions in 2010. Yang et al. [22] and Wang et al. [23] calculated the embodied SO_2_ emissions in China’s interregional trade, respectively, and found the embodied emissions were far more than the actual emissions. Li et al. [24] calculated the embodied PM_2.5_ emissions of 21 sectors in the Jing-Jin-Ji region and found that transport and storage, electricity, hot water, gas and water production and supply and services were the key sectors emitting PM_2.5_ from the production-, consumption- and income-based perspectives, respectively. Xie et al. [25] calculated the embodied air pollutant emissions in China from 1995 to 2009 and found that China’s total air pollutant emissions have an obvious turning point in 2001. Chang et al. [26] coupled the EIOA model with the life cycle inventory model and calculated the embodied air pollutant emissions footprint of buildings in China.

Key sectors can be identified according to the direct or embodied pollutant emissions of each sector. Scholars generally believe that the sectors with a large proportion of direct emissions are the key sectors on the production side, while the sectors with a large proportion of embodied emissions is the key sectors on the consumption side [9,24]. In addition, the hypothetical extraction method (HEM) on the basis of the IOA model is often used to analyze the effect of sector separation on total output and further to decompose sectoral linkages [27]. Usually, the key demand sectors and output sectors can be identified through the net linkage that reflects the difference between the forward linkage and backward linkage [28,29,30]. Wang et al. [8] applied HEM to analyze the flows of embodied air pollutant emissions of China in 2010 and found that the construction, machinery manufacturing and service sectors were the key sectors in terms of demand embodied emissions, and power and gas, nonmetal products and metal mining, smelting and pressing sectors were the key sectors in terms of output embodied emissions. He et al. [31] analyzed the changes in linkages of 2002 and 2010 amongst inter industrial air pollutant emissions and found that the transport equipment and electrical equipment sectors were the key demand embodied emissions and the power and gas sectors were responsible for the growing SO_2_ emissions. Zhang et al. [12] revealed the linkages of SO_2_ and NO_x_ emissions between sectors from 2012 to 2017 and found that the metal melting sector and equipment manufacturing sector were the largest pollutant output emission sector and demand emission sector, respectively. Structural path analysis (SPA) based on EIOA can excavate intricate sectoral interrelationships by extracting supply chain paths step by step and trace the key emission sectors and paths [32,33,34]. Yang et al. [22] and Qi et al. [35] applied SPA to analyze the variation of SO_2_ emissions embodied in Chinese supply chains during 2002–2012 and 2005–2015, respectively, and found that the dominant SO_2_ emission sectors differed under production and consumption perspectives. Song et al. [36] adopted SPA to extract the critical sectors and supply chains of air pollutions and found that the sectors of power and heat, metals smelting and nonmetallic mineral products were major contributors to production-based emissions, and the sectors of construction, equipment and services were major contributors to consumption-based emissions.

Ecological network analysis (ENA) has advantage in analyzing the structure distribution and functional relationships within the ecosystem and the whole system robustness [37]. It has been widely used in many aspects, such as energy [38,39], water [40], carbon emissions [41], etc. By combing ENA and EIOA, Yang et al. [42] and Wakeel et al. [43] respectively analyzed the mutual interactions and control relationship among sectors in China and India with respect to embodied PM_2.5_ and identified the dominant sectors. Song et al. [44] conducted network control analysis (NCA) and network utility analysis (NUA) on China’s SO_2_ emissions in 2010 and 2015 and found that most sectors had control over transportation equipment, electronic equipment and construction; almost all sectors had dependence on power and heat; and exploitative relationships were predominant. Complex network theory can analyze the geometric properties of networks and find key nodes [45]. By combing EIOA and complex network analysis (CNA), scholars analyzed the interrelationships among different sectors in the industrial chain network formed by the embodied carbon emissions or O_3_ and identified the key sectors on the basis of degree centrality and betweenness centrality [46,47].

A literature review shows that the EIOA model can reflect the transfer relationship of air pollutant emissions among different economic sectors in detail. The key sectors can be sought according to the proportion of emissions, and the critical path can be obtained by SPA. Combined with the EIOA model, ENA from the perspective of ecological metabolism and CAN from the perspective of complex network can be used to obtain pollutant emission characteristics and identify functional relationships among sectors. In fact, ENA and CAN are complementary to each other in the analysis process and conclusion due to their different analysis principles. However, the existing studies only use one method to study pollutant emissions from one perspective, which affects the comprehensiveness and reliability of the results to a certain extent. In addition, most of the existing studies focus on the analysis of a single air pollutant, rather than the common analysis of different air pollutants. In fact, different pollutant emissions are closely related to various sectors, and there is a certain overlap in the key pollution source sectors. Through the joint analysis of different air pollutants, we can more systematically obtain the emission reduction path of air pollutants. This study makes up for the deficiency of existing studies and makes contributions in the following three aspects: First, we analyzed the system characteristics and key sectors of air pollutant emissions from the perspective of ENA and CAN. Second, considering that NO_x_ and PM_2.5_ are the air pollutants that need to be significantly reduced in China’s 14th Five-Year Plan but there is a lack of PM_2.5_ data from various sectors, we conducted a joint analysis on NO_x_ and particulate matter (PM). Third, we determined the key pollution source sectors from the supply side and the demand side, respectively, so as to provide decision support for more efficient pollutant emission reduction.

The rest of this paper is organized as follows: Section 2 presents the methods and explains the data sources. Section 3 analyzes the sectoral characteristics, intersectoral correlation characteristics and overall system characteristics of air pollutant emissions in China, and identifies the key sectors of air pollutant emissions. Section 4 discusses emission reduction measures in key sectors. Section 5 summarizes the research of this paper.

## 2. Method and Data

### 2.1. Method

#### 2.1.1. EIOA

Environmental input–output table is a physical input–output table formed by expanding environmental elements on the basis of Leontief’s input–output table. Its framework is shown in Table 1.

Assuming that there are *n* sectors, according to the horizontal equilibrium relationship of the EIOA model, the following equation can be obtained:

(1) AX+Y−im=X

where $A={\left[a_{ij}\right]}_{n\times n}$ is direct consumption coefficient matrix, and the element $a_{ij}=\frac{Z_{ij}}{X_{j}}$ means that the inputs from sector *i* are transformed into the outputs of sector *j* with the support of production technologies; *X*, *Y* and *im* are *n* × 1 total outputs, final outputs and imports column vector, respectively.

In order to eliminate the influence of import, the direct consumption coefficient matrix of domestic products $A^{d}$ is defined [48]; then, Formula (1) can be modified as:

(2) $$X={\left(I-A^{d}\right)}^{-1}Y=LY$$

where *I* is the *n* × *n* identity matrix; $L={\left(I-A^{d}\right)}^{-1}$ is the Leontief inverse matrix, and the elements $l_{ij}$ represent the amount of output from sector *i* that is directly and indirectly required to produce one unit of final demand from sector *j*.

Similarly, according to the vertical equilibrium relationship of the EIOA model, the following equation can be obtained:

(3) $$X^{T}A^{\prime }+V=X^{T}$$

where $A^{\prime }={\left[\frac{Z_{ij}}{x_{i}}\right]}_{n\times n}$ is the direct distribution coefficient matrix; *V* is 1 × *n* added value row vector. Formula (3) can be modified as:

(4) $$X^{T}=V{\left(I-A^{\prime }\right)}^{-1}=VG$$

where $G={\left[g_{ij}\right]}_{n\times n}={\left(I-A^{\prime }\right)}^{-1}$ is the Ghosh inverse matrix.

The row vector of direct discharge coefficient is defined as follows:

(5) $$E={\left[e_{j}\right]}_{1\times n}={\left[\frac{P_{j}}{X_{j}}\right]}_{1\times n}\;$$

where$\;P_{j}$ represents the direct pollutant emissions of the *j*th sector; $e_{j}$ represents the emission of pollutants from the unit output of *j*th sector. Combining the Leontief inverse matrix and Ghosh inverse matrix, we can get:

(6) $$C={\left[c_{ij}\right]}_{n\times n}=\hat{E}L\;$$

(7) $$B={\left[b_{ij}\right]}_{n\times n}=G\hat{E}$$

where $\hat{E}$ is the diagonal matrix form of E. The influence coefficient ($PFL_{i}$) and sensitivity coefficient ($PBL_{j}$) of pollutant emissions are defined as follows:

(8) $$PFL_{i}=\frac{\sum_{j=1}^{n}c_{ij}}{\frac{1}{n}\sum_{i=1}^{n}\sum_{j=1}^{n}c_{ij}}\left(i=1,2\ldots ,n\right)$$

(9) $$PBL_{j}=\frac{\sum_{i=1}^{n}b_{ij}}{\frac{1}{n}\sum_{i=1}^{n}\sum_{j=1}^{n}b_{ij}}\;\left(j=1,2\ldots ,n\right)$$

$PFL_{i}>1$ means that the impact of the production of sector *i* on other sectors exceeds the average impact level; otherwise, it means that the impact of the production of f sector *i* is less than or equal to the average impact level. Similarly, $PBL_{j}>1$ means that the sensitivity of the *j*th sector is higher than the average sensitivity level; otherwise, it means that the sensitivity of the *j*th sector is less than or equal to the average sensitivity level.

**Figure 1:**
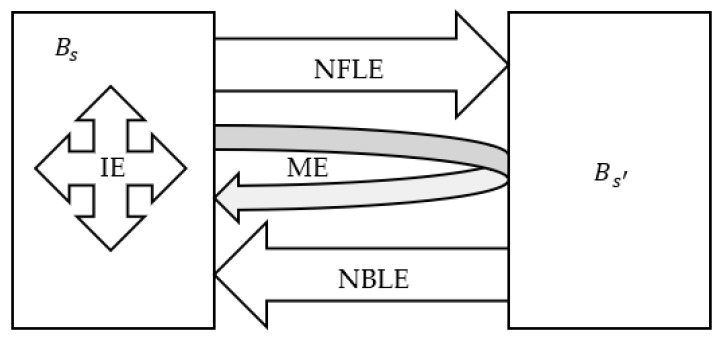
Analysis of air pollutant emission linkages. (IE: internal emission; ME: mixed emission; NBLE: net backward linkage emission; NFLE: net forward linkage emission).

Based on the hypothesis extraction method, the emissions of air pollutant can be decomposed, and the dependence relationship between industrial sectors can be obtained, as shown in Figure 1. $B_{s}$ represents a block of target sectors in the economy, while $B_{s}^{\prime }$ represents the remaining blocks. For each block $B_{s}$, which can be made up of either a single sector or several sectors, Formula (2) can be described as:

(10) $$\left[\begin{array}{c} X_{s}\\ X_{s^{\prime }} \end{array}\right]=\left[\begin{array}{cc} {A^{d}}_{s,s} & {A^{d}}_{s,s^{\prime }}\\ {A^{d}}_{s^{\prime },s} & {A^{d}}_{s^{\prime },s^{\prime }} \end{array}\right]\left[\begin{array}{c} X_{s}\\ X_{s^{\prime }} \end{array}\right]+\left[\begin{array}{c} Y_{s}\\ Y_{s^{\prime }} \end{array}\right]=\left[\begin{array}{cc} \Delta_{s,s} & \Delta_{s,s^{\prime }}\\ \Delta_{s^{\prime },s} & \Delta_{s^{\prime },s^{\prime }} \end{array}\right]\left[\begin{array}{c} Y_{s}\\ Y_{s^{\prime }} \end{array}\right]$$

where the vector of total output $X=\left[\begin{array}{c} X_{s}\\ X_{s^{\prime }} \end{array}\right]$. The direct consumption coefficient matrix of domestic products $A^{d}=\left[\begin{array}{cc} {A^{d}}_{s,s} & {A^{d}}_{s,s^{\prime }}\\ {A^{d}}_{s^{\prime },s} & {A^{d}}_{s^{\prime },s^{\prime }} \end{array}\right]$; Leontief inverse matrix $L=\left[\begin{array}{cc} \Delta_{s,s} & \Delta_{s,s^{\prime }}\\ \Delta_{s^{\prime },s} & \Delta_{s^{\prime },s^{\prime }} \end{array}\right]$; final outputs $Y=\left[\begin{array}{c} Y_{s}\\ Y_{s^{\prime }} \end{array}\right]$.

Net backward linkage emissions (*NBLE*) reflect the net emission imports of industrial sectors, and net forward linkage emissions (*NFLE*) reflect the net emission exports. They can be calculated as follows:

(11) $$NBLE_{s}=P_{s^{\prime }}\Delta_{s^{\prime },s}Y_{s}$$

(12) $$NFLE_{s}=P_{s}\Delta_{s,s^{\prime }}Y_{s^{\prime }}$$

where the row vector of direct discharge coefficient $E=\left[\begin{array}{cc} P_{s} & P_{s^{\prime }} \end{array}\right]$.

#### 2.1.2. Ecological Network Analysis

In order to explore the emission relationship of embodied pollutants among sectors, it can be seen from Table 1 that the equation is as follows [49]:

(13) $$P+\varepsilon Z=\varepsilon \hat{X}\;$$

(14) $$\varepsilon =P{\left(\hat{X}-Z\right)}^{-1}$$

where $Z={\left[z_{ij}\right]}_{n\times n}$ is the monetary value flow matrix in the input-output table; $\hat{X}$ is a diagonal matrix composed of the total economic output of each sector; $\varepsilon ={\left[\varepsilon_{j}\right]}_{1\times n}$ is the row vector of embodied pollutant emission intensity; and $\varepsilon_{j}$ represents the direct and indirect increase in pollutant emissions of the entire economic system for each additional unit of output of sector *j*. The embodied pollutant emission flow $f_{ji}$ from sector *i* to sector *j* can be calculated by multiplying the monetary value flow matrix by the embodied pollutant emission intensity matrix:

(15) $$\begin{array}{ccc} f_{11} & \ldots & f_{n1}\\ \vdots & f_{ji} & \vdots \\ f_{1n} & \ldots & f_{nn} \end{array}=\hat{\varepsilon }Z$$

where $\hat{\varepsilon }$ is the diagonal matrix transformed from the row vector of embodied pollutant emission intensity.

Under steady-state conditions, incoming and outgoing throughflows are equal: $T_{i}^{in}=T_{i}^{out}=T_{i}$. Since it is not possible to obtain detailed and reliable input data for a complex air pollutant emission system, we use only the output data to calculate the throughflows for each component. Then, the total flow *T_i_* of sector *i* and the total network flow can be obtained through Formulas (16) and (17):

(16) $$T_{i}=\sum_{j=1}^{n}f_{ji}+P_{i}$$

(17) $$T=\sum_{i=1}^{n}T_{i}\;\;$$

According to the ratio of intersector flow to total flow, the input-driven and output-driven dimensionless direct flow intensity matrices are *G* = [*g_ij_*]*_n_*_×*n*_ and *G*′ = [*g_ij_*′]*_n_*_×*n*_, respectively. Where *g_ij_* = *f_ij_*/*T_j_* is the output-oriented flows from node *j* to node *i*; *g_ij_*′ = *f_ij_*/*T_i_* is the input-oriented flows from node *j* to node *i*. Then, the dimensionless total flow intensity matrices *N* and *N*′ can be obtained:

(18) $$N={\left[n_{ij}\right]}_{n\times n}=G^{0}+G^{1}+G^{2}+\cdots +G^{k}+\cdots ={\left(I-G\right)}^{-1}\;$$

(19) $$N^{\prime }={\left[{n_{ij}}^{\prime }\right]}_{n\times n}={\left(G^{\prime }\right)}^{0}+{\left(G^{\prime }\right)}^{1}+{\left(G^{\prime }\right)}^{2}+\cdots +{\left(G^{\prime }\right)}^{k}+\cdots ={\left[I-\left(G^{\prime }\right)\right]}^{-1}$$

where *I* is the identity matrix, $n_{ij}$ represents the integral dimensionless value of $g_{ij}$, which is calculated using a Leontief inverse matrix. *G*^0^ represents the self-feedback effect generated by the flow through each sector; *G*^1^ represents the direct flow transferred and exchanged between sectors; the indirect flow intensity of different path lengths is expressed by the higher power of *G*, and *G^m^* (*m* ≥ 2) represents the indirect flow intensity of path length *m* between sectors.

NCA can identify the dependency or control relationship between sectors. The control matrix *CX* = [*cx_ij_*]*_n_*_×*n*_, where:

(20) $$cx_{ij}=\{\begin{array}{c} 1-\frac{n_{ij}}{n_{ji}^{\prime }},\;\;\frac{n_{ij}}{n_{ji}^{\prime }}<1\\ 0\;\;,\;\;\;\;\;\;\;\;\;\;\frac{n_{ij}}{n_{ji}^{\prime }}>1 \end{array}$$

$cx_{ij}=0$ means that the pollutant output effect from sector *j* to sector *i* is greater than the input effect from sector *i* to sector *j*, and sector *j* controls sector *i*. $cx_{ij}<1$ means that the output effect from sector *j* to sector *i* is less than the input effect from sector *i* to sector *j*, and sector *j* is dependent on sector *i*. The closer $cx_{ij}$ is to 1, the lower the output effect of sector *j* on *i* is than the input effect of sector *i* on *j*, and the more obvious the dependence of sector *j* on *i* is.

*NUA* is an ecological network method to analyze the relationship between paired departments. Positive utility represents the acquisition of resources or elements, while negative utility represents the loss of resources or elements. The direct relationship between sectors can be represented by the dimensionless direct effect matrix *D* = [*d_ij_*]*_n_*_×*n*_, where *d_ij_* = (*f_ij_* − *f_ji_*)/*T_i_* represents the utility means the utility from node *j* to node *i*. Then the dimensionless comprehensive effect matrix *U* is as follows:

(21) $$U={\left[u_{ij}\right]}_{n\times n}=D^{0}+D^{1}+D^{2}+\cdots +D^{k}+\cdots ={\left(I-D\right)}^{-1}\;$$

where *I* is the identity matrix,$\;u_{ij}$ represents the integral dimensionless value of $d_{ij}$,which is calculated using a Leontief inverse matrix, and the matrix *U* represents the flows of integrated relations between any pair of sectors in the network. The identity matrix (*D*^0^) reflects the self-feedback of flows through each sector, the matrix *D*^1^ reflects the direct flow utilities between any two sectors in the network, *D*^2^ represents the indirect flow utilities that pass along two steps, and *D^k^* (*k* ≥ 2) reflects the indirect flow utilities along *k* steps.

The symbols of the elements in *U* constitute the matrix *sjgn*(*U*), in which each element is recorded as *su_ij_.* For the symbol pair (*su_ij_*, *su_ji_*): (+, −) indicates that sector *i* utilizes or exploits sector *j* (i.e., receives more utility than it transfers to node *j*), but node *j* suffers (receives less utility than it transfers to node *i)*; (−, +) represents that sector *i* is exploited or controlled by sector *j*; (−, −) represents the competition between the two sectors, and although this is a negative situation in the short term, competition may be essential to promote the system’s long-term development because it encourages both nodes to improve their efficiency and to look for ways in which they can cooperate; (+, +) represents the mutually beneficial symbiosis of sectors; (0, 0) indicates neutrality. Let *S*_+_(*U*) and *S*_−_(*U*) respectively represent the number of positive and negative signs in *U*; then, the network symbiosis index is as follows:

(22) $$MI=\frac{\;S_{+}\left(U\right)}{\;S_{-}\left(U\right)}$$

*MI* > 1 indicates that the positive effect between sectors in the system is greater than the negative effect, and the overall performance is a symbiotic system in which sectors promote each other; *MI* < 1 indicates that the system is in an unstable state of competitive inefficiency.

Ecosystem robustness is affected by the balance between the efficiency of the network structure and the resilience of the system. Ascendancy *A* represents the structurally constrained part of the system and can be calculated as follows:

(23) $$A=T\times AMI=T\times \left[\sum_{i=1}^{n}\sum_{j=1}^{n}\left(\frac{f_{ij}}{T}\right)\log_{2}\left(\frac{f_{ij}T}{T_{i}T_{j}}\right)\right]$$

where *AMI* is the average mutual information index, reflecting the average degree of mutual restriction of material, energy or information in the system [50]. The resilience *R* reflects the system redundancy and can be calculated as follows:

(24) $$R=T\times H_{R}=T\times \left[-\sum_{i=1}^{n}\sum_{j=1}^{n}(\frac{f_{ij}}{T})\log_{2}\left(\frac{f_{ij}^{2}}{T_{i}T_{j}}\right)\right]$$

where *H_R_* is the degree of freedom. In the process of system evolution, ascendancy and resilience are relatively independent forces. Only when the balance between them is in a reasonable range can the system be sustainable. The system robustness index *SR* can be defined as follows:

(25) $$SR=-\alpha \log_{2}\alpha \;$$

where α is the ratio of ascendancy *A* to the development capacity *C*. The latter can be calculated as follows:

(26) $$C=T\times D_{R}=T\times \left[-\sum_{i=1}^{n}\sum_{j=1}^{n}\left(\frac{f_{ij}}{T}\right)\log_{2}(\frac{T_{j}}{T})\right]$$

where *D_R_* is the diversity index. The larger the *SR*, the better the stability of the ecosystem and the stronger its sustainable development ability. 

#### 2.1.3. Complex Network Analysis 

Based on the embodied pollutant emission flow matrix, the complex network model of intersectoral flow can be expressed as: G=(V,E,W), where $V=\left(v_{1},v_{2},\ldots ,v_{n}\right)$ represents the node set in the network; *E* represents the edge set; and the weight *W* of the edge represents the embodied pollutant emissions between sectors. In order to further explore the flow relationship of embodied pollutant emissions between sectors, we set thresholds according to the emission flow. If the flow is less than the threshold, the edge is deleted. If a sector is not connected to any other sector, we delete the sector from the network. We set thresholds and remove some edges according to the following formula:

(27) $$\tilde{f_{ij}}=\{\begin{array}{c} 1,f_{ij}>f_{j}\\ 0,f_{ij}<f_{j} \end{array},f_{j}=\frac{1}{n}\sum_{i=1}^{n}f_{ij}$$

$\tilde{f_{ij}}=1$ means that there is an edge from sector *j* to sector *I*, and $\tilde{f_{ij}}=0$ means that there is no edge from sector *j* to sector *i*. Degree centrality $S_{i}$, that is, the sum of the edges of sector *i*, can be defined to comprehensively reflect the local information of sector *i*. According to the direction of the flow, node degree can be divided into in-degree $S_{i}^{in}$ and out-degree $S_{i}^{out}$:

(28) $$S_{i}^{in}=\sum_{j=1\left(j\neq i\right)}^{n}\tilde{f_{ij}},\;\;S_{i}^{out}=\sum_{j=1\left(j\neq i\right)}^{n}\tilde{f_{ji}}\;$$

Closeness centrality is an indicator of the closeness degree of a sector to other sectors, reflecting the independence and effectiveness of a sector. If a sector is connected with most sectors through a short distance, it indicates that the sector has a high degree of closeness to the center and strong uncontrolled ability. Closeness centrality includes in-closeness centrality $C_{c}{\left(i\right)}^{in}$ and out-closeness centrality $C_{c}{\left(i\right)}^{out}$:

(29) $$C_{c}{\left(i\right)}^{in}=\frac{n-1}{\sum_{j=1}^{n}d_{ij}},\;C_{c}{\left(i\right)}^{out}=\frac{n-1}{\sum_{j=1}^{n}d_{ji}}$$

where $d_{ij}$ is the distance between two sectors, i.e., the number of edges in the shortest path from node *j* to node *i*.

Betweenness centrality can reflect the ability of a sector to control other sectors in the network. The higher the betweenness centrality is, the more important the sector plays as a bridge for the association between other sectors. It is defined as follows:

(30) $$C_{g}\left(k\right)=\sum_{i<j}\frac{g_{ij}\left(k\right)}{g_{ij}}\;$$

where $C_{g}\left(k\right)$ is the betweenness centrality of sector *k*, $g_{ij}$ is the number of shortest paths between sector *i* and sector *j* and $g_{ij}\left(k\right)$ is the number of shortest paths through sector *k* between sector *i* and sector *j*.

The average shortest path *L* is defined as the average distance to represent the transfer efficiency of the network, and its formula is as follows:

(31) $$L=\frac{\sum_{i\neq j}d_{ij}}{\frac{n\left(n-1\right)}{2}}\;$$

The sectoral clustering coefficient $C_{i}$ is the ratio of the number of edges connected to the sector to the maximum number of possible edges in the network. The global clustering coefficient *C* describes the tightness of the network, which is the average of all the sectoral clustering coefficients. Their calculation formulas are as follows:

(32) $$C_{i}=\frac{2E_{i}}{m_{i}\left(m_{i}-1\right)},\;C=\frac{1}{n}\sum_{i}C_{i}$$

where $m_{i}$ is the number of sectors directly connected to sector *i*, and $E_{i}$ is the number of edges connected with sector *i*.

Network density can reflect the density of nodes’ interconnecting edges in the network. The higher the network density is, the closer the connections between nodes are. Network density can be calculated as follows:

(33) $$D_{en}=\frac{N_{0}}{n\left(n-1\right)}$$

where *N*_0_ is the actual number of connections in the network.

Network correlation degree can reflect the network accessibility degree. The less mutually unreachable node pairs, the higher the network correlation degree. The formula is as follows:

(34) $$CN=1-\frac{V}{\frac{n\left(n-1\right)}{2}}\;$$

where *V* is the number of mutually unreachable node pairs.

#### 2.1.4. Structural Path Analysis

SPA is used to track and extract the complex production process chain motivated by a given final demand. It can quantify the environmental transmission in the upstream process to reveal how emissions are transferred from producers to consumers and then determine the important path with the greatest potential for environmental improvement. The Leontief inverse matrix can be decomposed by using Taylor’s expansion:

(35) $$L^{d}={\left(I-A^{d}\right)}^{-1}=I+A^{d}+{\left(A^{d}\right)}^{2}+\cdots +{\left(A^{d}\right)}^{r}+{\left(A^{d}\right)}^{>r}\;$$

Then, the total pollutant emissions caused by domestic final demand can be expressed as:

(36) $$P=EL^{d}Y=E{\left(I-A^{d}\right)}^{-1}Y=EY+EA^{d}Y+E{\left(A^{d}\right)}^{2}Y+E{\left(A^{d}\right)}^{>2}Y$$

In the above formula, each item on the right represents a specific production layer. The first item PL(0)=EY can be regarded as the pollutant emissions directly caused by the final demand, the second item $PL\left(1\right)=EA^{d}Y$ can be regarded as the pollutant emissions indirectly attributed to inter-sectoral intermediate input and the third item $PL\left(2\right)=E{\left(A^{d}\right)}^{2}Y$ or higher level $E{\left(A^{d}\right)}^{>2}Y$ represents the pollutant emissions generated by the input of components in the relevant supply chain. In fact, there is a practical limit to the number of supply chains that can be extracted. Therefore, we only calculate the emissions generated at the first three layers referring to previous studies [22,36].

### 2.2. Data Sources and Processing

In this study, we mainly use two data sets: China’s 2018 input–output (IO) table [51] and NO_x_ and PM emissions of each industrial sector in 2018 [52]. Since the classification of sectors in the IO table is different from that of environmental statistics, we merge the sectors involved in the IO table into 27 industrial sectors, as shown in Table 2.

## 3. Results

### 3.1. Sectoral Pollutant Emissions

**Figure 2:**
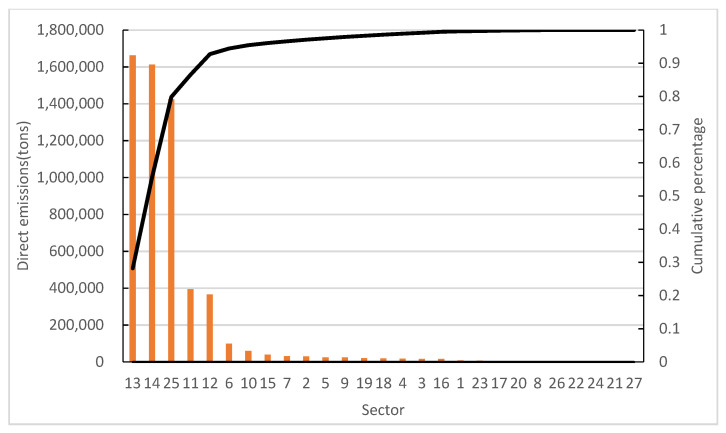
Direct emissions of of NO_x_.

**Figure 3:**
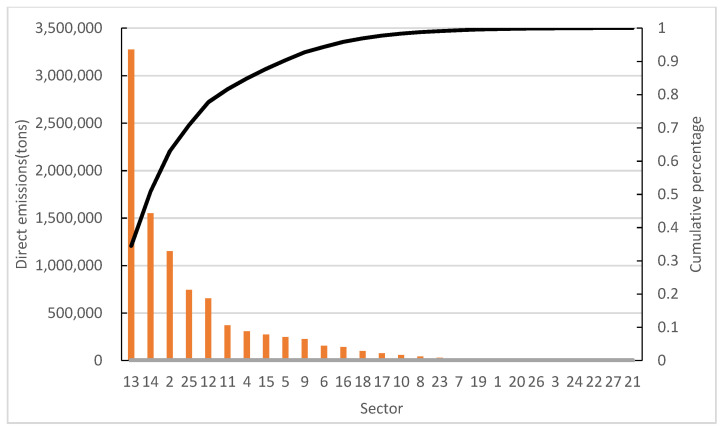
Direct emissions of PM.

The direct emissions of NO_x_ and PM of 27 sectors are shown in Figure 2 and Figure 3, respectively. Sectors 13, 14 and 25 are the key direct NO_x_ emission sectors, accounting for 80% of total emissions. Sectors 2, 12, 13, 14 and 25 are the key direct PM emission sectors, accounting for 78% of total emissions.

**Figure 4:**
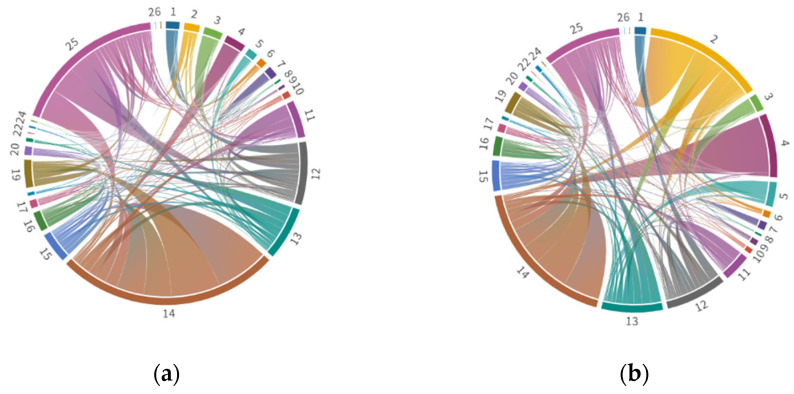
Flow structure of embodied pollutants between industrial sectors. (**a**) NO_x_; (**b**) PM.

Figure 4 shows the flow structure of embodied NO_x_ and PM emissions between industrial sectors. Directional chord diagram can visualize the flows to and from each pair of entity (chords are thicker at one end than the other). For example, the top three embodied emissions export–import flows pairs of NO_x_ exist in sector 14 to sector 15, sector 14 to sector 19 and sector 25 to sector 14 with 346,865.5888 tons, 324251.9577 tons and 198,843.3736 tons, respectively. Sector 15 and sector 19 are the largest demanders of intermediate products of sector 14, and sector 14 is the biggest requester of sector 25, which is consistent with their sectoral characteristics with the demand for raw materials. Therefore, sectors 12, 13, 14 and 25 are the key embodied NO_x_ emission sectors; sectors 2, 12, 13, 14 and 25 are the key embodied PM emission sectors.

### 3.2. Sectoral Influence and Sensitivity

**Figure 5:**
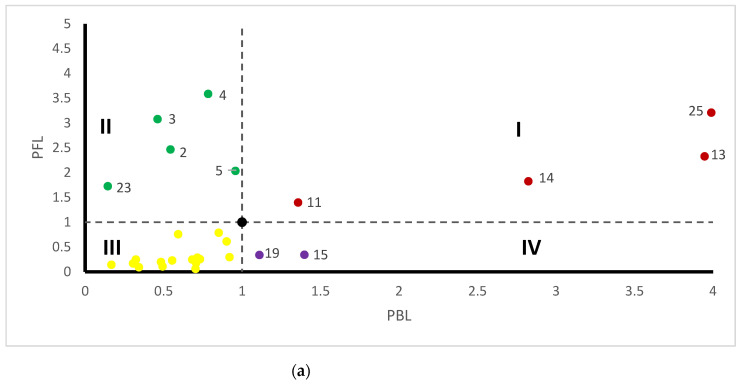

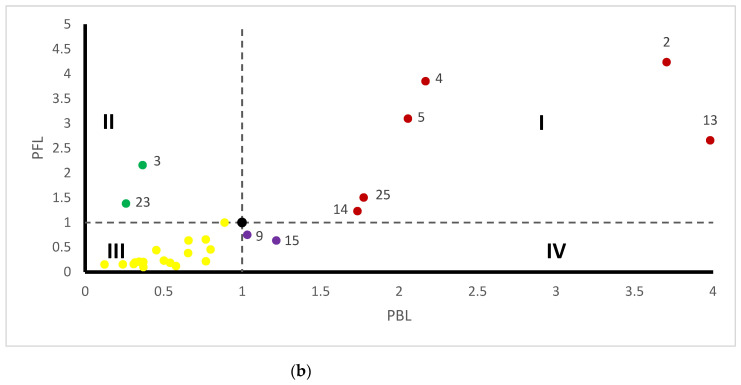
The influence and sensitivity coefficients of NO_x_ and PM in 27 sectors. (**a**) NO_x_; (**b**) PM. The influence and sensitivity coefficients of the sectors in the quadrant III are less than the threshold value 1, which is not the focus of our research. Therefore, the sectors represented by the yellow scatter is not marked.

The influence and sensitivity coefficients of NO_x_ and PM in 27 sectors are shown in Figure 5, where “I/II/III/IV” represent four different quadrants. The influence and sensitivity coefficients of the sectors in the quadrant I are greater than the threshold value 1, which are generally identified as key sectors. In addition, attention should also be paid to sectors in the quadrant II and quadrant IV. The influence coefficient of sectors in quadrant II is greater than 1, indicating that the increase in emissions in one sector in this quadrant has a significant impact on the increase in emissions in other sectors. The sensitivity coefficient of sectors in quadrant IV is greater than 1, indicating that the increase in emissions of one sector in this quadrant is greatly affected by the increase in emissions of other sectors. 

### 3.3. Net Forward and Backward Linkage Emissions

Table 3 shows the net forward and backward linkage emissions of 27 sectors. Sectors 18, 20, 19, 16 and 17 have large net backward linkage emissions; therefore, the efficiency of the use of products in these sectors should be improved, thereby indirectly promoting pollutant emission reductions. Sectors 14, 13 and 12 have large net forward linkage emissions in both NO_x_ and PM, indicating that these sectors have more net pollutant emissions and need to strengthen emission reduction.

### 3.4. Network Control Analysis

**Figure 6:**
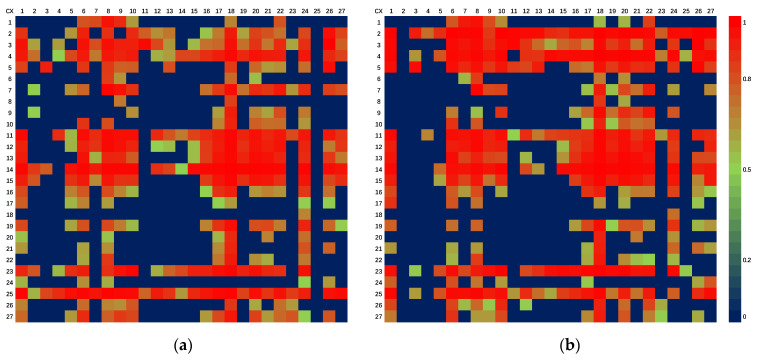
Pairwise control relationships between sectors of pollutant emissions. (**a**) NO_x_; (**b**) PM.

The pairwise control relationships between sectors are shown in Figure 6. From the perspective of columns, it can be seen that the red accounts for the majority of sectors 6, 8, 18, 20 and 22, indicating that food and tobacco; garments, shoes, hats, leather and eiderdown; transportation equipment; communication equipment; computers and other electronic equipment; and other manufactured products have the same characteristics, and they play the role of the dependent rather than the controller in the atmospheric pollutant metabolic network. On the contrary, sectors 2, 3, 11, 23 and 25 mainly play controlling roles; therefore, more air pollutants are discharged due to meeting the production activities of the industrial sectors.

Furthermore, the production and supply of electricity and heat, coal mining and washing respectively occupy an absolute control position in the NO_x_ and PM ecological network system (i.e., they all control the other 26 sectors). Agriculture, forestry, animal husbandry, fishery and their services; food and tobacco; garments, shoes, hats, leather and eiderdown; and transportation equipment are the most serious dependents on production and supply of electricity and heat, which are all over 95%. For example, they are with the amount of 96.08%, 95.17%, 96.67% and 97.72% in the NO_x_ ecological network system in 2018, respectively. Therefore, we should reduce their pollutant emissions while strengthening their own consumption savings and improving output efficiency.

### 3.5. Network Utility Analysis

**Figure 7:**
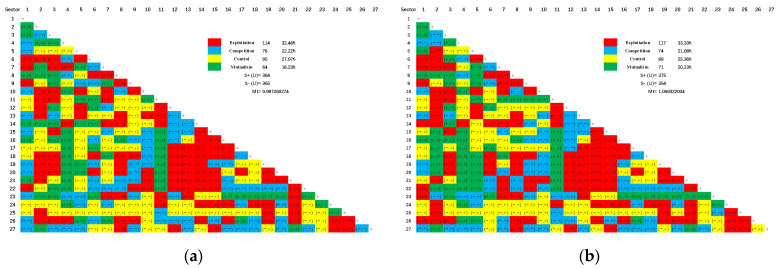
Pairwise utility relationships between sectors. (**a**) NO_x_; (**b**) PM.

Figure 7 shows the relationships of exploitation, competition, control and symbiosis between sectoral pairs. Different from energy and water, nitrogen oxides and particulate matter are negative ecological factors in the environment, and exploitation and control relationships can be regarded as positive absorption and absorption. As can be seen from Figure 6, the exploitation and control relationships dominate the two networks, accounting for more than 55%. The proportions of competition are greater than those of symbiosis in two networks. Therefore, China’s industrial air pollutant emission system is a metabolic system dominated by the “exploitation” activities between paired sectors. The calculation results of symbiosis index of NO_x_ and PM are 0.9973 and 1.059, respectively. Both of them are close to 1, indicating that China’s air pollutant emission system is a healthy metabolic system on the whole. 

Besides, most mutualism relationships are related to petroleum, coking and nuclear fuel processing, followed by metal mining and processing. The competition relationships exist in all sectors, with most relationships related to water production and supply, and paper making and printing. Most sectors involve many pairs of exploitative relationships, and the most relationships are related to nonmetallic mineral products, metal smelting and rolling, and instruments and apparatuses. Production and supply of electricity and heat, special equipment, repair services for metal products, machinery and equipment are the main contributors, which are dominated by control relationship due to these sectors being the pillar industry of industrial production.

However, there are too few mutualism relationships between sectors, so it is necessary to increase the mutualism relationships between sectors to maintain the mutually beneficial symbiotic state of the system, so that the paired sectors can promote and progress each other in the communication, so as to contribute greater benefits to the system.

### 3.6. Robustness Analysis

**Figure 8:**
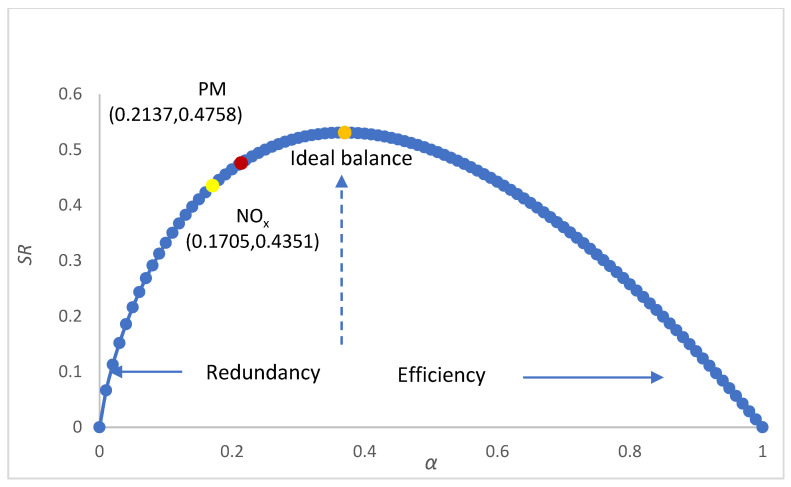
Robustness of air pollutant emission systems.

Figure 8 shows the robustness analysis result. PM has higher robustness than NO_x_, but both of them are in the range of large redundancy and low efficiency. This indicates that key sectors do not play prominent roles in the system structure, which to some extent reduces the coordinated emission reduction efficiency of sectors. Therefore, it is necessary to identify key sectors to formulate precise emission reduction strategies.

### 3.7. Critical Path Analysis

When producing the finally consumed products, about 26–30% direct embodied emissions among total embodied emissions are generated. Larger indirect embodied emissions are induced by the upstream stages of the supply chains. It can be seen from Table 4 that in the four decomposition items, the first EY of the two pollutants in sectors 6, 11, 12, 13, 14, 23 and 25 is greater than the other three, indicating that the direct pollutant emissions mainly meet the final demand and export account for a relatively high proportion. The second $EA^{d}Y$ of the two pollutants in sectors 1, 10, 15, 16, 17, 18, 19, 21, 22, 24, 26 and 27 is greater than the other three, indicating that the pollutant emissions caused by meeting the first intermediate demand for final use and export account for a relatively high proportion. The fourth item $E{\left(A^{d}\right)}^{>2}Y$ of the two pollutants in sector 4 is larger than the other three items, indicating that the pollutant emissions caused by meeting the third intermediate demand for final use and export account for a relatively high proportion.

By using SPA, we can extract the key supply chains of NO_x_ and PM emissions and reveal how pollutant emissions are transferred from producers to consumers. Table 5 and Table 6 respectively list the top 30 critical paths driven by the final demand category for the emissions of two pollutants. To distinguish the sectors at PL0, PL1 and PL2, we give them the marks of “0”, “1” and “2”. The cumulative contributions of the top 30 paths to total NO_x_ and PM emissions are 49.95% and 46.04%, respectively. In addition, over 50% of emissions are attributed to PL1. This reveals that only focusing on the emission-intensive sectors is not enough to achieve effective air pollution control. The emissions from the upstream sectors should also be controlled to mitigate those of the downstream and the ending sectors. Overall, 22 of the top 30 paths are found to be common to NO_x_ and PM emissions, and the common critical paths of NO_x_ and PM are marked with the same color in Table 5 and Table 6. Taking NO_x_ as a reference, the paths ranked 1, 2, 3, 4, 5, 6, 7, 8, 9, 10, 11, 12, 13, 14, 15, 16, 17, 19, 20, 21, 24 and 26 are common paths. 

We selected the sectors (PL0) with the top 20% of emissions in the 30 key paths as the key sectors on the demand side, and their embodied emissions to meet the final domestic demand are relatively large. The key demand-side sectors of NO_x_ include sectors 12, 13, 14, 18, 19 and 25, and those of PM include sectors 2, 9, 12, 13, 14, 18 and 25. It can be seen that sectors 12, 13, 14, 18 and 25 are common key sectors of NO_x_ and PM. Since the PL1 and PL2 layers involve a small number of sectors, we select all these sectors as the key sectors on the supply side. They meet the final domestic demand through the path of PL2 → PL1 → PL0. The key supply-side sectors of NO_x_ include sectors 11, 13, 14, 18, 19 and 25, and those of PM include sectors 6, 9, 12, 13, 14 and 16. Sectors 13 and 14 are the common key supply-side sectors of NO_x_ and PM.

### 3.8. Complex Network Analysis

**Figure 9:**
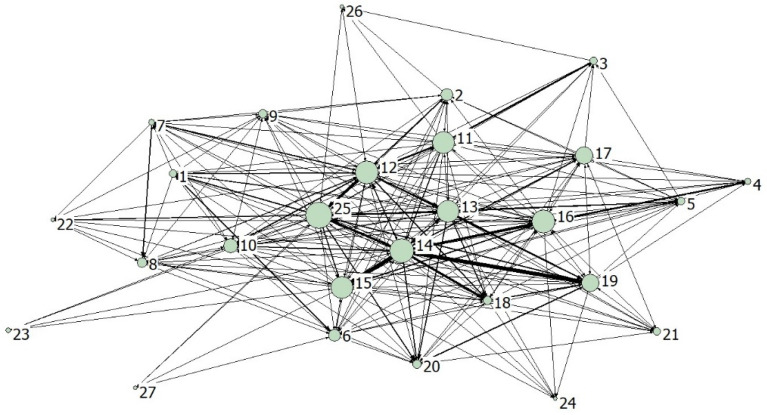
The correlation networks of NO_x_ emissions between industrial sectors.

**Figure 10:**
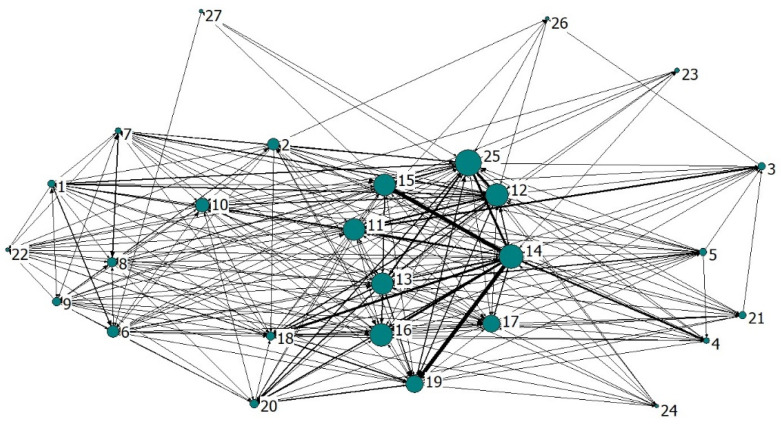
The correlation networks of PM emissions between industrial sectors.

The complex networks of NO_x_ and PM are shown in Figure 9 and Figure 10, respectively, containing 294 and 357 weighted directed edges. The 27 nodes in the figure represent 27 industrial sectors, the node size represents the out-degree of the sector, and the edge weight represents the embodied pollutant emissions between sectors. The coarser the edge represents the deeper the correlation between the sectors.

The degree, closeness and betweenness of NO_x_ and PM in each sector are shown in Table 7 and Table 8, respectively. Out-degree and in-degree can be used to describe the “initiator” and “acceptor” of air pollutant emission, and promote the responsible distribution of pollutant emissions. The result shows that sectors 12, 13, 14, 15 and 25 are the key initiators of NO_x_ and PM, and their out-degrees occupy the top 5 among 27 sectors. For China, a large economic country, many sectors have produced a large number of pollutant emissions while maintaining their own production and operation. As key sectors, the initiators have significant impacts on the pollutant emission system, and their emission reduction should be strengthened. Sectors 10, 12, 13, 16 and 18 are the key acceptors, and they should be focused on in the formulation of emission reduction target, so that emission reduction can achieve the high efficiency target of “one drives many”.

Closeness centrality reveals the importance of a sector according to the distance. The shorter the distance between a sector and other sectors, the more convenient the sector is in information exchange and resource exchange, and the more profound the impact on the pollutant emissions from other sectors. Similar to degree centrality, out-closeness reflects the influence of a sector as pollutant emission “source”. The main source sectors of NO_x_ include sectors 11, 12, 13, 14, 15, 16 and 25, and those of PM include sectors 12, 13, 15, 16 and 25. In-closeness centrality reflects the influence of a sector as pollutant emission “sink”. The main sink sectors of NO_x_ include sectors 10, 12, 13, 16 and 18, and those of PM include sectors 10, 12, 13, 16, 18 and 19.

Betweenness centrality reflects the role of a sector as a “bridge” in the pollutant emission network, including the impact of receiving pollutants from the related upstream sector, and the impact of transferring pollutants to the downstream sectors. The main “bridge” sectors of NO_x_ include sectors 1, 12, 14, 17 and 19, and those of PM include sectors 8, 12, 13, 15, 16 and 25.

The network densities of NO_x_ and PM are 0.336 and 0.413, and their network correlation degrees are 0.553 and 0.641, respectively. The network densities less than 0.5 and correlation degrees greater than 0.5 mean that the two pollutant emission flow relationships between sectors are both relatively loose on the whole, but the degrees of mutual accessibility between sectors are relatively high.

The small-world property of the network indicates that nonadjacent nodes arrive through a small distance, which is formally characterized by the two indicators of average shortest path and clustering coefficient. High clustering coefficient and short average path indicate that the network has small world and good connectivity. The average shortest paths of NO_x_ and PM metabolism networks in China’s industrial sectors are 1.688 and 1.641, respectively, that is, each sector only needs 1.688 or 1.641 steps to reach other sectors and generate pollutant emission links, indicating that the air pollutant metabolism network has strong transmission capacity and connectivity. The clustering coefficients of NO_x_ and solid networks are 0.647 and 0.705, respectively, indicating that the adjacent sectors are closely linked. The large clustering coefficients and the short average paths confirm that the air pollutant metabolism network conforms to the nature of a small world network, which provides information for the overall structural characteristics and evolution of the network. Changes in key sectors will greatly affect the system functions of the whole network. During the implementation of emission reduction, this attribute can be used to focus on key sectors to promote the collaborative emission reduction of other sectors, so as to improve the environmental performance of China’s industrial system.

### 3.9. Identification of Key Sectors of Air Pollutant Emissions

The key sectors determined by different methods are different. Most methods can identify the key sectors on the supply side and the demand side. Indicators without direction can be regarded as two-way discrimination indicators. Table 9 summarizes the discrimination indicators and basis from the supply side and the demand side.

We summarize the key supply-side and demand-side sectors of NO_x_ and PM emissions obtained by the above seven methods, and the results are shown in Table 10 and Table 11, respectively. It can be seen that sectors 12, 13, 14, 25, 15, 16, 18 and 19 are the key joint supply-side and joint demand-side sectors for both NO_x_ and PM emissions. In particular, the first four sectors are identified as key supply-side sectors and key demand-side sectors at least four times in the seven methods.

## 4. Discussion

Different sectors have different positions in the supply chain of pollutant emissions, especially key sectors undertake the responsibility of supply-side or demand-side emission reduction. The emission reduction strategies of different categories of key sectors are discussed as follows:

(1) Sectors 12 (chemical products), 13 (nonmetallic mineral products), 14 (metal smelting and rolling), 25 (production and supply of electricity and heat), 15 (metal products), 16 (general equipment), 18 (transportation equipment) and 19 (electrical machinery and equipment) are the key joint supply-side and joint demand-side sectors for both NO_x_ and PM emissions. For these sectors, on the one hand, it is necessary to strengthen the emission reduction of pollutants in the production process and reduce their transfer of pollutants to other sectors. On the other hand, it is necessary to reduce the consumption demand for them and avoid causing more emissions of pollutants. Taking the sector of production and supply of electricity and heat as an example, measures such as increasing pollutant disposal devices in the production process and switching to high-quality and low-pollution energy sources should be taken to reduce pollutant emissions. At the same time, the production utilization rate of the sector should be increased, so as to reduce the demand for its products to a certain extent.
(2) Sectors 2 (coal mining and washing), 4 (metal mining and processing), 5 (nonmetal mining and processing), 17 (special equipment) and 23 (scrap wastes) are the key joint supply-side sectors for both NO_x_ and PM emissions and the key demand-side sectors for NO_x_ or PM emissions. For these sectors, we should adopt the same emission reduction measures as the sectors in category (1), both strengthening the emission reduction of pollutants in the production process and reducing the demand for them. Taking the sector of coal mining and washing as an example, attention should be paid to the treatment of pollutants in the process of mining and washing, while reducing the consumption of washed coal.
(3) Sectors 3 (oil and gas extraction) and 20 (communication equipment, computers and other electronic equipment) are the key joint supply-side sectors for both NO_x_ and PM emissions, but neither the key demand-side sectors for NO_x_ emissions nor those for PM emissions. For these two sectors, we should pay attention to the emission reduction of pollutants in the production process and appropriately expand sectoral production to meet the consumption demand of more sectors for their products.
(4) Sector 10 (paper making, printing and cultural and educational sports articles) is the key joint demand-side sector for both NO_x_ and PM emissions, but neither the key supply-side sector of NO_x_ emissions nor that of PM emissions. We should pay attention to reduce consumption demand by saving products of this sector and improving its production efficiency.

## 5. Conclusions

In this paper, based on the EIOA model, we conducted production-based and consumption-based accountings of China’s NO_x_ and PM emissions of 27 industrial sectors in 2018, analyzed their sectoral influence and sensitivity coefficients, and obtained the system characteristics and key sectors of air pollutant emissions in combination with ENA and CAN. SPA is further employed to reveal how the disparities between production and consumption attribution of emissions arise, and extract the critical sectors and supply chains for air pollution co-control in China. The main conclusions are as follows:

(1) The relationships between sectors in the system are different. Some sectors depend on other sectors, while others control other sectors. For example, food and tobacco; garments, shoes, hats, leather and eiderdown; transportation equipment; communication equipment, computers and other electronic equipment; and other manufactured products mainly play the role of the dependent in the atmospheric pollutant metabolic network, while sectors such as metal mining and processing; oil and gas extraction; petroleum, coking and nuclear fuel processing; scrap wastes and production and supply of electricity and heat play controlling roles. China’s industrial air pollutant emission system is a metabolic system dominated by the “exploitation” activities between paired sectors. “Exploitation” and “control” account for 59.55% and 58.69% of NO_x_ and PM ecological network systems, respectively. This system is a healthy metabolic system on the whole, but it has certain redundancy and low efficiency in structure. It needs to strengthen the management of key sectors and implement precise emission reduction strategies.
(2) The direct embodied emissions in PL0 account for only 26–30% for two pollutants, with over half of embodied emissions attributed to PL1. The top 30 paths contribute nearly 50% of total embodied emissions. The paths “from sector 13 (nonmetallic mineral products) to final demand” and “from sector 14 (metal smelting and rolling) to final demand” are the top two critical paths for the emissions of both two pollutants.
(3) Using seven methods, we identified the key joint supply-side or joint demand-side sectors for both NO_x_ and PM emissions, including the sectors of chemical products, nonmetallic mineral products, metal smelting and rolling, production and supply of electricity and heat, metal products, general equipment, transportation equipment, electrical machinery and equipment, etc. This indicates that the joint emission reduction of NO_x_ and PM can be achieved through common emission reduction paths or strategies.
(4) The joint key sectors are divided into four types, and targeted emission reduction strategies are proposed respectively, such as strengthening pollutant emission reduction in the production process for key supply-side sectors and enhancing production efficiency and reducing dependence on their sector products for key demand-side sectors. For example, sectors 12 (chemical products), 13 (nonmetallic mineral products), 14 (metal smelting and rolling), 25 (production and supply of electricity and heat), 15 (metal products), 16 (general equipment), 18 (transportation equipment) and 19 (electrical machinery and equipment) are the key joint supply-side and joint demand-side sectors for both NO_x_ and PM emissions. We should strengthen the emission reduction of pollutants in the production process and reduce their transfer of pollutants to other sectors, reduce the consumption demand for them and avoid causing more emissions of pollutants on the meanwhile.

The findings of this study provide useful implications for policy making on air pollution control with focus on the key sectors and supply chains. The adopted methodology has applicability in addressing similar problems, such as emissions of other pollutants and water pollutant discharges, etc. One limitation of this study is that the detailed data on industrial sectoral air pollutant emissions in 2018 are unavailable to match the 2018 I–O table of China. Once sufficient data support is possible, we can attain updated results as obtained in this work. Future work will be extended to involve emissions of greenhouse gases (CO_2_, CH_4_, N_2_O, O_3_, etc.) and other air pollutants (CO, hydrocarbon, aerosols, etc.) to investigate the ways towards co-control of both global warming and air pollution.

## Figures and Tables

**Table 1:** Framework for environment extended input–output table.

Input		Output						
		Intermediate Outputs				Final Outputs	Imports	Total Outputs
		Sector 1	Sector 2	…	Sector *n*			
Intermediate inputs	Sector 1	${\left[Z_{ij}\right]}_{n\times n}$				$Y_{i}$	*im*	$X_{i}$
	Sector 2							
	…							
	Sector *n*							
Initial inputs		$V_{j}$						
Total inputs		$X_{j}$						
Pollutant emissions		$P_{j}$						

**Table 2:** The 27 sectors.

Code	Sector
1	Agriculture, forestry, animal husbandry, fishery and their services
2	Coal mining and washing
3	Oil and gas extraction
4	Metal mining and processing
5	Nonmetal mining and processing
6	Food and tobacco
7	Textile
8	Garments, shoes, hats, leather and eiderdown
9	Wood products and furniture
10	Paper making, printing and cultural and educational sports articles
11	Petroleum, coking and nuclear fuel processing
12	Chemical products
13	Nonmetallic mineral products
14	Metal smelting and rolling
15	Metal products
16	General equipment
17	Special equipment
18	Transportation equipment
19	Electrical machinery and equipment
20	Communication equipment, computers and other electronic equipment
21	Instruments and apparatuses
22	Other manufactured products
23	Scrap wastes
24	Repair services for metal products, machinery and equipment
25	Production and supply of electricity and heat
26	Gas production and supply
27	Water production and supply

**Table 3:** Net forward and backward linkage emissions of 27 sectors.

NO_x_				PM			
Sector	*NFLE*	Sector	*NBLE*	Sector	*NFLE*	Sector	*NBLE*
14	704,339.3	18	292,619.4	14	677,105.4	18	418,808.2
25	483,099	20	228,117.9	2	492,433.3	20	335,420.4
13	194,441.2	19	223,729.6	13	383,155.3	19	310,809.2
12	112,341.8	16	184,770.8	25	252,448.4	16	242,459.3
11	88,720.17	17	149,286.4	12	201,312.2	17	210,711.6
7	14,946.67	15	94,746.71	4	155,230.4	12	144,720.1
2	12,546.95	12	91,457.87	11	83,523.94	6	136,264.4
15	10,595.92	6	88,235.77	15	74,044	15	119,414.1
6	9756.83	8	68,162.81	5	58,574.49	8	94,830.41
4	8593.784	1	44,194.35	16	35,326.27	25	75,964.62
10	7334.44	10	40,588.29	9	23,362.01	1	65,202.42
3	6602.122	9	31,093.42	6	15,258.47	10	64,564.98
5	5754.561	14	26,963.58	23	14,400.61	14	61,737.7
19	5201.843	7	21,388.12	7	12,113.11	9	45,109.49
16	3948.499	21	20,697.64	17	11,112.37	13	43,787.4
1	3616.58	13	20,568.04	10	7005.522	11	39,338.5
23	3104.147	11	13,449.99	19	4742.183	7	32,482.07
9	2441.152	25	10,131.59	1	4556.709	21	31,198.5
17	852.9738	22	8039.262	18	3556.211	22	11,655.33
18	692.8305	27	8033.826	8	2877.44	26	8950.903
20	508.0852	26	4510.926	3	1813.147	27	8362.931
26	280.0338	24	4053.829	20	1271.143	3	6220.059
8	232.1496	3	2796.319	26	705.7357	24	5570.295
24	83.51876	5	2264.007	24	579.5835	5	3174.59
22	68.30131	2	331.5896	22	315.6887	2	363.3055
21	13.11352	23	22.30991	27	86.80502	23	28.54703
27	6.610978	4	−131.716	21	76.91585	4	−161.872

**Table 4:** Pollutant emission structure path of China’s industrial sector in 2018.

Sector	NO_x_				PM			
	EY	$EA^{d}Y$	$E{\left(A^{d}\right)}^{2}Y$	$E{\left(A^{d}\right)}^{>2}Y$	EY	$EA^{d}Y$	$E{\left(A^{d}\right)}^{2}Y$	$E{\left(A^{d}\right)}^{>2}Y$
1	2658.112	16,447.6	12,054.95	7385.15	3349.087	20,913.45	18,302.37	11,932.13
2	134.2351	157.3592	98.4579	51.38412	5268.36	915.1351	242.5101	94.92712
3	1407.256	1279.118	788.97	374.3102	386.4761	3859.777	1137.095	612.2146
4	−41.3584	−72.2117	−32.7624	−15.3499	−747.061	−113.587	−51.302	−26.1265
5	1059.539	1214.397	547.1008	267.9812	10,784.83	1644.303	884.4518	457.1683
6	51,495.92	33,012.75	27,713.06	18,436.67	80,533.23	50,728.71	40,858.26	28,656.45
7	6072.175	7496.798	6540.556	4519.325	4921.022	9080.244	9149.946	6855.944
8	2197.497	16,761.63	17,472.98	13,482.27	27,237.47	22,619.68	23,747.88	19,728.14
9	8209.53	11,523.34	9514.872	6113.984	78,565.84	35,622.67	19,667.06	11,146.75
10	14,519.31	17,551.76	12,382.17	7215.251	13,868.18	24,897.82	19,072.65	11,605.64
11	58,453.09	8590.778	4033.416	2211.68	55,029.56	23,636.59	11,420.21	3960.165
12	56,245.38	55,750.91	32,390.35	17,320.73	100,789.5	87,415.76	54,004.69	29,856.18
13	91,473.25	25,868	9405.598	3951.166	180,252.3	55,873.43	18,310.57	7202.84
14	83,182.8	33,802.37	13,804.8	5785.343	79,966.47	51,447.12	22,407.36	9771.46
15	7167.376	48,561.9	25,887.51	11,742.65	50,085.44	55,262.5	37,390.15	18,636
16	8313.683	70,256.87	54,948	31,035.98	74,380.53	88,747.46	76,204.3	46,629.9
17	4248.359	52,006.62	44,223.7	26,284.19	55,346.73	72,246.05	62,330.72	39,498.91
18	11,451.3	93,412.61	85,046.75	55,125.3	58,778.08	133,970	124,148.4	83,562.96
19	8212.423	98,694.84	63,467.38	32,592.74	7486.733	119,128.4	92,101.22	50,782.28
20	2379.943	59,207.73	62,625.66	45,452.97	5954.212	82,189.72	90,450.67	68,256.3
21	22.68602	7075.147	5892.172	3610.807	133.0622	10,359.59	8700.388	5538.214
22	162.043	3439.965	2196.884	1176.734	748.9626	4410.94	3318.547	1895.573
23	42.37658	14.50085	6.27815	3.017331	196.5914	29.10271	10.21571	5.013116
24	116.6438	1500.003	1190.263	675.0549	809.4566	1771.97	1657.451	1029.661
25	131,250.4	40,277.65	14,707.51	6203.138	68,586.28	58,734.74	26,190.05	11,210.34
26	1120.365	2143.038	1214.097	656.4371	2823.522	3987.606	3016.55	1208.441
27	21.01284	4403.547	1997.674	866.5778	275.9077	2929.602	2700.989	1430.715

Notes: Grey in the Table 4 represents the highest proportion of the four decomposition items.

**Table 5:** The top 30 paths for China’s NO_x_ emissions start with the final demand category and end up in a sector.

Rank	PL	Contribution	NO_x_ Emissions	Path		
				Sector (PL0)	Sector (PL1)	Sector (PL2)
1	0	6.33%	131,250.47	S25		
2	0	4.41%	91,473.27	S13		
3	0	4.01%	83,182.81	S14		
4	1	3.36%	69,656.66	S19	S14	
5	0	2.82%	58,453.06	S11		
6	1	2.78%	57,634.79	S18	S14	
7	0	2.71%	56,245.36	S12		
8	1	2.49%	51,697.31	S16	S14	
9	0	2.48%	51,495.93	S6		
10	1	1.83%	37,888.37	S25	S25	
11	1	1.67%	34,720.8	S15	S14	
12	1	1.57%	32,503.15	S17	S14	
13	1	1.05%	21,733.01	S14	S14	
14	1	1.03%	21,283.8	S20	S14	
15	1	0.96%	19,821.18	S12	S12	
16	1	0.91%	18,917.53	S20	S13	
17	2	0.88%	18,199.06	S19	S14	S14
18	1	0.88%	18,187.75	S12	S25	
19	1	0.84%	17,357.91	S13	S13	
20	2	0.82%	17,070.34	S18	S18	S14
21	1	0.74%	15,394.35	S19	S13	
22	2	0.73%	15,058.13	S18	S14	S14
23	0	0.70%	14,519.32	S10		
24	1	0.69%	14,361.01	S18	S13	
25	2	0.65%	13,506.86	S16	S14	S14
26	0	0.55%	11,451.32	S18		
27	1	0.55%	11,426.06	S20	S25	
28	2	0.53%	10,937.32	S25	S25	S25
29	2	0.51%	10,578.13	S19	S19	S14
30	1	0.49%	10,235.46	S6	S25	

**Table 6:** The top 30 paths for China’s PM emissions start with the final demand category and end up in a sector.

Rank	PL	Contribution	PM Emissions	Path		
				Sector (PL0)	Sector (PL1)	Sector (PL2)
1	0	5.59%	180,252.28	S13		
2	0	3.12%	100,789.48	S12		
3	0	2.50%	80,533.03	S6		
4	0	2.48%	79,966.46	S14		
5	0	2.43%	78,565.88	S9		
6	0	2.30%	74,380.61	S16		
7	0	2.13%	68,586.31	S25		
8	1	2.08%	66,963.31	S19	S14	
9	0	1.82%	58,778	S18		
10	1	1.72%	55,406.28	S18	S14	
11	0	1.72%	55,346.67	S17		
12	0	1.71%	55,029.54	S11		
13	0	1.55%	50,085.45	S15		
14	1	1.54%	49,698.38	S16	S14	
15	1	1.16%	37,277.86	S20	S13	
16	1	1.15%	37,051.53	S25	S2	
17	1	1.10%	35,518.78	S12	S12	
18	1	1.06%	34,204.55	S13	S13	
19	1	1.03%	33,378.28	S15	S14	
20	1	0.97%	31,246.38	S17	S14	
21	1	0.94%	30,335.27	S19	S13	
22	1	0.88%	28,299.02	S18	S13	
23	0	0.84%	27,237.37	S8		
24	1	0.74%	23,781.16	S9	S9	
25	1	0.65%	20,892.69	S14	S14	
26	1	0.63%	20,460.85	S20	S14	
27	1	0.61%	19,798.96	S25	S25	
28	2	0.54%	17,495.38	S19	S14	S14
29	1	0.54%	17,408.94	S18	S18	
30	1	0.53%	16,985.35	S12	S2	

**Table 7:** The degree, closeness and betweenness of NO_x_ metabolic network.

Sector	Out-Degree	In-Degree	Out-Closeness	In-Closeness	Betweenness
1	5	7	0.5200	0.4727	42.7500
2	7	9	0.5778	0.5000	0.0000
3	4	5	0.5417	0.4561	2.5000
4	4	8	0.5200	0.4906	0.0000
5	3	8	0.5098	0.4906	0.0000
6	7	10	0.5778	0.5098	2.7500
7	5	6	0.5000	0.4643	23.0000
8	4	6	0.5200	0.4561	15.2000
9	4	10	0.5000	0.5000	10.0833
10	10	14	0.5909	0.5532	10.6667
11	17	6	0.7429	0.4643	6.5690
12	22	17	0.8387	0.5909	116.3857
13	20	14	0.8125	0.5532	36.1952
14	18	11	0.7429	0.5200	45.1190
15	19	11	0.7429	0.5200	25.1262
16	17	13	0.7429	0.5417	5.2833
17	14	11	0.6667	0.5200	44.5000
18	5	14	0.5098	0.5532	0.0000
19	11	11	0.6341	0.5200	48.9262
20	5	11	0.4906	0.5200	0.9167
21	6	8	0.5652	0.4960	0.0000
22	0	6	0.2000	0.4906	0.0000
23	2	1	0.4643	0.3514	0.0000
24	1	5	0.5098	0.4407	0.0000
25	26	9	1.0000	0.4815	16.9452
26	0	3	0.2000	0.4000	1.0833
27	0	2	0.2000	0.4333	0.0000

Notes: Grey in the Table 7 represents the key sectors of NO_x_ identified based on the corresponding centrality indicators.

**Table 8:** The degree, closeness and betweenness of PM metabolic network.

Sector	Out-Degree	In-Degree	Out-Closeness	In-Closeness	Betweenness
1	6	10	0.5306	0.5652	3.2253
2	7	9	0.7647	0.5778	16.7483
3	4	5	0.5532	0.5098	5.5493
4	4	8	0.5200	0.5652	0.1429
5	3	8	0.5909	0.5652	1.5833
6	7	10	0.6047	0.6190	13.2825
7	5	6	0.5000	0.5652	1.1690
8	4	6	0.5532	0.5909	27.3571
9	4	10	0.6190	0.6047	10.1739
10	10	14	0.6190	0.6842	15.9106
11	17	6	0.7879	0.5200	7.2926
12	22	17	0.8387	0.7647	93.2329
13	20	14	0.8387	0.6842	51.4978
14	18	11	0.7429	0.6047	18.2904
15	19	11	0.8125	0.6047	22.8623
16	17	13	0.8125	0.6341	25.7603
17	14	11	0.6842	0.6047	12.2047
18	5	14	0.5306	0.6500	8.0314
19	11	11	0.6842	0.6500	16.3192
20	5	11	0.5532	0.6047	1.3663
21	6	8	0.5652	0.5532	2.0930
22	0	6	0.3662	0.5417	0.2857
23	2	1	0.5200	0.3714	0.0000
24	1	5	0.5200	0.5000	1.2776
25	26	9	1.0000	0.5417	77.3435
26	0	3	0.5200	0.4262	0.0000
27	0	2	0.2000	0.5098	0.0000

Notes: Grey in the Table 8 represents the key sectors of PM identified based on the corresponding centrality indicators.

**Table 9:** The identification methods of key sectors.

Method	Supply-Side	Demand-Side
1	Sectors with high proportion of direct emissions	Sectors with high proportion of embodied emissions
2	Sectors with influence coefficient greater than 1	Sectors with sensitivity coefficient greater than 1
3	Sectors with large net forward linkage emissions	Sectors with large net backward linkage emissions
4	Sectors with large out-degree	Sectors with large in-degree
5	Sectors with large out-closeness centrality	Sectors with large in-closeness centrality
6	Sectors (PL0) with top 20% of emissions in 30 key paths	Sectors (PL1 and PL2) in 30 key paths
7	Sectors with large betweenness centrality	Sectors with large betweenness centrality

**Table 10:** The key supply-side sectors of NO_x_ and PM emissions.

Supply																	Joint Key Sectors
Sector	Method																
	NO_x_								PM								
	1	2	3	4	5	6	7	Frequency	1	2	3	4	5	6	7	Frequency	
S1							○	1								0	
S2		○						1	●	●						2	**√**
S3		○						1		●						1	**√**
S4		○						1		●						1	**√**
S5		○						1		●						1	**√**
S6								0						●		1	
S7								0								0	
S8								0							●	1	
S9								0						●		1	
S10								0								0	
S11		○			○	○		3								0	
**S12**				○	○		○	**3**	●			●	●	●	●	**5**	**√△**
**S13**	○	○		○	○	○		**5**	●	●		●	●	●	●	**6**	**√△**
**S14**	○	○		○	○	○	○	**6**	●	●		●		●		**4**	**√△**
S15				○	○			2				●	●		●	3	**√△**
S16			○		○			2			●		●	●	●	4	**√△**
S17			○				○	2			●					1	**√**
S18			○			○		2			●					1	**√△**
S19			○			○	○	3			●					1	**√△**
S20			○					1			●					1	**√**
S21								0								0	
S22								0								0	
S23		○						1		●						1	**√**
S24								0								0	
**S25**	○	○		○	○	○		**5**	●	●		●	●		●	**5**	**√△**
S26								0								0	
S27								0								0	

Notes: “○” and “●” represent the key departments on the supply side of NO_x_ and PM, respectively; “**√**” represents the joint key sector on the supply side of the above two pollutants; “**√△**” represents the joint key sectors on the supply side and demand side of the two pollutants; the corresponding sectors in bold format represent the key sectors identified in China’s industrial air pollutant system.

**Table 11:** The key demand-side sectors of NO_x_ and PM emissions.

Demand																	Joint Key Sectors
Sector	Method																
	NO_x_								PM								
	1	2	3	4	5	6	7	Frequency	1	2	3	4	5	6	7	Frequency	
S1							○	1								0	
S2								0	●	●	●			●		4	
S3								0								0	
S4								0		●						1	
S5								0		●						1	
S6								0								0	
S7								0								0	
S8								0							●	1	
S9								0		●				●		2	
S10				○	○			2				●	●			2	**√**
S11		○	○					2								0	
**S12**	○		○	○	○	○	○	**6**	●		●	●	●	●	●	**6**	**√△**
**S13**	○	○	○	○	○	○		**6**	●	●	●	●	●	●	●	**7**	**√△**
**S14**	○	○	○			○	○	**5**	●	●	●			●		**4**	**√△**
S15		○						1		●					●	2	**√△**
S16				○	○			2				●	●		●	3	**√△**
S17							○	1								0	
S18				○	○	○		3				●	●	●		3	**√△**
S19		○				○	○	3					●			1	**√△**
S20								0								0	
S21								0								0	
S22								0								0	
S23								0								0	
S24								0								0	
**S25**	○	○	○			○		**4**	●	●	●			●	●	**5**	**√△**
S26								0								0	
S27								0								0	

Notes: “○” and “●” represent the key departments on the demand side of NO_x_ and PM, respectively; “**√**” represents the joint key sector on the demand side of the above two pollutants; “**√△**” represents the joint key sectors on the supply side and demand side of the two pollutants; the corresponding sectors in bold format represent the key sectors identified in China’s industrial air pollutant system.

## Data Availability

The data used to support the findings of this study are available from the corresponding author upon request.
